# Enhancing mung bean hydration using the ultrasound technology: description of mechanisms and impact on its germination and main components

**DOI:** 10.1038/srep38996

**Published:** 2016-12-19

**Authors:** Alberto Claudio Miano, Jessica da Costa Pereira, Nanci Castanha, Manoel Divino da Matta Júnior, Pedro Esteves Duarte Augusto

**Affiliations:** 1 Department of Agri-food Industry, Food and Nutrition (LAN), Luiz de Queiroz College of Agriculture (ESALQ), University of São Paulo (USP), Piracicaba, SP, 13418900, Brazil

## Abstract

The ultrasound technology was successfully used to improve the mass transfer processes on food. However, the study of this technology on the grain hydration and on its main components properties was still not appropriately described. This work studied the application of the ultrasound technology on the hydration process of mung beans (*Vigna radiata*). This grain showed sigmoidal hydration behavior with a specific water entrance pathway. The ultrasound reduced ~25% of the hydration process time. In addition, this technology caused acceleration of the seed germination – and some hypothesis for this enhancement were proposed. Moreover, it was demonstrated that the ultrasound did not change both structure and pasting properties of the bean starch. Finally, the flour rheological properties proved that the ultrasound increased its apparent viscosity, and as the starch was not modified, this alteration was attributed to the proteins. All these results are very desirable for industry since the ultrasound technology improves the hydration process without altering the starch properties, accelerates the germination process (that is important for the malting and sprouting process) and increases the flour apparent viscosity, which is desirable to produce bean-based products that need higher consistency.

The hydration process is an important step before many others grain process such as cooking, germination, extraction, malting and fermenting. It is a discontinuous and time spender process, being limiting in the industrial processing. Therefore, its improvement is very desirable.

In fact, many works have used higher soaking temperatures to enhance this process^1^,^2^,^3^,^4^,^5^,^6^,^7^,^8^. However, the use of high temperatures can change the properties of the grains components and alter their nutritional composition. In addition, temperatures can bring additional use of water for the heating system, as well as the amount of energy. Consequently, other technologies are being studied to improve the hydration process, being the ultrasound technology one of the most promising.

The ultrasound technology has been successfully used in many mass transfer processes in food, such as in drying, extraction, osmotic dehydration, desalting and hydration. The enhancement of the mass transfer by ultrasound is attributed to its direct and/or indirect effects, which depend on the food properties (porosity and water activity)^9^. The direct effects are related to the ultrasonic wave traveling through the food, which causes the expansion and compression of the medium. These effects are the called “sponge effect” (when the cells or the food matrix is compared to a sponge squeezed and released repeatedly) and the inertial flow (mass flow due to the wave propagation). The indirect effects are related to changes in the product structure caused by the acoustic cavitation, resulting in cell and matrix disruption, and then creating micro cavities (or micro channels) that improve the mass transfer^9^,^10^,^11^.

In fact, the ultrasound technology was successfully used to enhance the hydration process of foods. However, it was studied only for a small number of grains, such as sorghum grains^12^, navy beans^13^, chickpeas^14^, common beans^15^ and corn kernels^16^, as well as on the rehydration of other kind of food such as sea cucumber^17^. Even so, the application of this technology should still be studied, in special for grains, where the hydration process is the limiting step during the industrial processing.

Most importantly, once the positive effect of the ultrasound technology on the hydration process was already demonstrated for some foods, it is now necessary to conduct studies not only for further products, but also for those with different behaviors and purposes. Consequently, to demonstrate the involved mechanisms, and to evaluate the impact of this technology on selected properties and components of the product. For example, although the hydration of grains can show two behaviors (the downward concave shape (DCS) and the sigmoidal behaviors^18^ – see further discussion), several grains with the downward concave shape hydration behavior and only one with sigmoidal hydration behavior grain were studied. Thus, highlighting the importance of studying this technology in grains with the sigmoidal behavior.

In this work, the mung bean (*vigna radiata*) hydration assisted by the ultrasound technology was studied. It was used since it has a sigmoidal behavior and due to its importance as a food for direct consumption and from sprouting^19^,^20^. Consequently, this work aimed to study the effect of ultrasound technology not only on the hydration process of mung bean, but also on the possible structural and functional properties of its flour and starch.

## Results and Discussion

### Mung bean hydration behavior description

**Figure 1:**
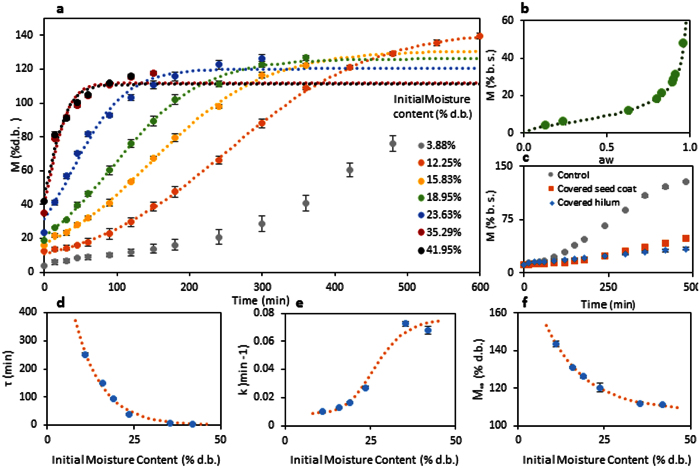
Mung bean hydration at 25 °C as function of its initial moisture content. The dots are the experimental values; the vertical bars are the standard deviation and the curves are the values obtained from the models. (**a**) Mathematical modeling using Kaptso *et al*. model (Equation 2) at different initial moisture contents. (**b**) Adsorption isotherm of mung bean (25 °C) (the data were modeled using the Oswin Model (Equation 1)). (**c**) Hydration (at 25 °C; 12.25% d.b) of mung bean under different treatments to explain the function of the seed coat and the hilum on the hydration kinetic. Effect of the initial moisture content on the Kaptso *et al*. parameters: (**d**) *τ* (Equation 3) (**e**) *k* (Equation 4) and (**f**) *M*_∞_ (Equation 5).

Depending on the seed coat permeability, grains can hydrate following two different behaviors: Downward concave shape (DCS) and Sigmoidal shape^21^. Figure 1a shows that mung bean has sigmoidal behavior during hydration under its normal (equilibrium with environment) initial moisture content (25 °C, 12.25% d.b.), similarly to other pulses such as Andean lupin^4^, Adzuki beans^3^,^21^,^22^, Cowpea^8^ and Italian Lima beans^23^. Further, its hydration behavior changes to the DCS when the initial moisture is increase.

The low permeability of the seed coat depends on its composition and its moisture content. The presence of callose, suberin and phenolic compounds in the seed coat can reduce its permeability^24^,^25^. In addition, the permeability of the seed coat increases when its moisture content is increased, changing the hydration behavior from sigmoidal shape to Downward Concave Shape (DCS) (Fig. 1a)^21^. This change on the seed coat permeability has two possible hypotheses. Firstly, when the moisture content of the bean is reduced, it can cause the shrinkage of cells, reducing the space between the seed coat and the cotyledon, and the closure of the hilum, avoiding the water entrance^26^. Secondly, the low moisture content may cause that the seed coat components pass from the rubbery state to the glassy state reducing its permeability^27^.

The state transition of the grain components is related to the grain’s water activity. Based on recent works^21^,^27^, there is a critical moisture content (due to a critical water activity value) when the hydration changes its behavior. According to Reid and Fennema^28^, the relation between the moisture content and the water activity (sorption isotherm; Fig. 1b) shows the different conditions that water has, depending on how the water is bound in the structure of the food, dividing the curve in three zones. Moreover, they state that the water activity when the water pass from the zone II to the zone III indicates the plasticization of the food structure, consequently the state transition. According to the sorption isotherm of mung bean (Fig. 1b, Equation 1) and this classification, the change of behavior would take place at approximately 0.83 of water activity, corresponding to approximately 23% d.b. of moisture content. This result agrees with Fig. 1, where the change of the hydration behavior (from sigmoidal to DCS) can be observe after ~23% d.b. of initial moisture content. In addition, it is interesting to highlight that the parameter values of the Oswin model (A and B)^29^ were similar to the obtained for Adzuky beans (A = 9.75 and B = 0.46)^21^, which means that the values could be similar for aleuro-amylaceous grains.


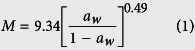


### Water pathway during mung bean hydration

**Figure 2:**
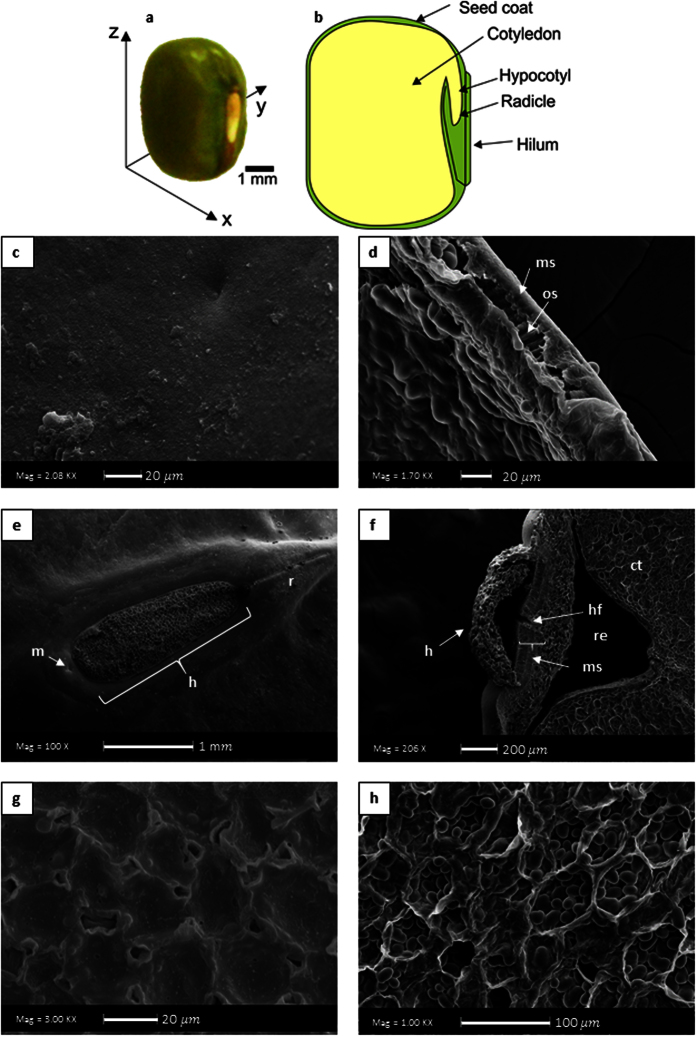
Morphology and microstructure (SEM, 20 kV; the magnifications are shown in the figures) of mung bean (*Vigna radiata*). (**a**) Real photo, scale bar and reference axes. (**b**) Representation of the longitudinal cut (xz plane) of the bean, with selected morphological structures. (**c**) External surface of seed coat. (**d**) Transversal cut of seed coat: ms. Macrosclereids, os. Osteosclereids. (**e**) h. Hilum, m. Micropyle, r. Raphe. (**f**) Transversal cut of the hilum: h. Hilum, re. Radicle space, ct. Cotyledon. hf. Hilar fissure. (**g**) Transversal cut of the radicle. (**h**) Cotyledon.

As all beans from the *fabaceae* family, mung bean has a complex structure (Fig. 2). Therefore, water may have a specific entrance route during the process and the mass transfer phenomena, as diffusion and capillarity, may take place together. The seed coat surface of this grain (Fig. 2c) does not have cracks or pores that permit the water to enter. In addition, the transversal cut of seed coat (Fig. 2d) shows the presence of the macroesclereids cells, common on this family of grains. Thus, all these structures give some degree of impermeability to the seed coat^30^. Further, osteosclereids cells are presented in the seed coat, which have large intercellular spaces probably contributing to the water lateral distribution^25^. Figure 2e shows the hilum, micropyle and raphe of the grain. The hilum is very porous, which probably allows the water to pass through. The transversal cut of the hilum (Fig. 2f) shows that this structure has direct contact with the radicle. In other words, this structure might cause the rapid hydration of the radicle to assure the activation of the germination process. The water would pass through the hilar fissure to the radicle, which has a porous structure (Fig. 2g) allowing the rapid water absorption. Figure 2e shows that the cotyledon is formed by a great quantity of starch covered by a protein matrix, which probably has a high affinity to water. In addition, the cotyledon structure has some intercellular spaces that can allow water to pass through. Therefore, once the water reaches the radicle and the cotyledon, they hydrate faster. However, this hydration might follow a specific path, starting from the radicle side until the rest of the grain.

The role of each grain structure in the water entrance is still controversial. For example the hilum is the principal water entrance for cowpeas^31^, while for Carioca beans and black beans, the entrance of water is by the micropyle, the raphe and the hilum^32^ despite this is more by the hilum. On the other hand, another works considered the hilum as the principal water entrance, as for black beans^33^ and for Andean lupin^4^. Although there is a probability that the water enters through the micropyle or raphe, the current work considers the hilum as the main water entrance. This was based on the observed microstructure (Fig. 2e and f), as the hilum has a significant larger area in comparison to the micropyle and raphe.

Furthermore, some treatments that describe the contribution of the seed coat and the hilum to the hydration process were performed (Fig. 1c), by covering (waterproofing) specific structures to know their participation in the process. When one of the structures (hilum or seed coat) was covered, the hydration rate was sharply reduced. When the hilum was covered, the hydration took place only by the seed coat; however, due to the low permeability of it, the process was very slow. Further, when the seed coat was covered, the hydration took place by the hilum. The hydration process was very slow despite the porosity of this structure. Due to the small area of this structure, the mass transfer through it is very low. In addition, it can be clearly seen that both structures have a synergic effect on the global hydration process (uncovered beans) since the sum of both hydration kinetics did not reach the uncovered bean hydration curve. It means that both structures work together to hydrate the whole bean. The water that enters by the hilum helps to accelerate the hydration of the seed coat, causing the change of its permeability, consequently accelerating the hydration process.

Similarly to previous works with soybean^34^, Andean lupin^4^ and adzuki beans^21^, the mung bean could have a similar water entrance pathway. The water probably enters through the hilum by capillarity and by the seed coat by diffusion depending on its moisture content.

With all the information explained above, the hydration pathway of this bean would be as follow: Firstly, the water mainly enters by the hilum (due to its porosity), hydrating the radicle slowly (due to its small area) to prevent drowning and assuring the metabolic activation. In addition, the osteoesclereids cells cause the lateral hydration of the bean (between the cotyledon and the seed coat) and the homogeneous distribution of water in the bean^25^. This first part is related to the initial lag phase of the process. Once the grain reaches approximately 23% d.b. of moisture content, the seed coat permeability changes drastically as it reaches the glassy transition moisture content, accelerating the hydration process. Finally, the water is distributed to the entire cotyledon until reaching the equilibrium moisture.

### Hydration process mathematical modeling

The hydration kinetics and the effect of the initial moisture content on the hydration behavior were mathematical modeled. Since mung bean has a sigmoidal behavior, Kaptso *et al*. model (Equation 2; ref. 8) was used at each moisture content obtaining a successful fit (Table 1). This model has explainable parameters with physical meaning. Therefore, they were useful to explain the behavior change of the hydration process.


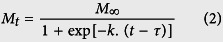


where *M*_*t*_ is the sample moisture content (% d.b.) at each time *t*; *M*_∞_ is the equilibrium moisture content; *τ* describes the necessary time to reach the inflection point of the curve, being thus related to the lag phase; and *k* is the water absorption rate kinetics parameter.

The parameter *τ* represents the lag phase duration. As the initial moisture content of the beans is increased, the value of this parameter exponentially decreases (Fig. 1d). This parameter tends to zero when the initial moisture content of the grain is higher than ~23% d.b., which means that the lag phase desapears and the sigmoidal hydration behavior turns into DCS behavior. This result was similar to Adzuki beans^21^. Consequently, an exponetial equation was used to model the effect of the initial moisture content on this parameters obtaining the Equation 3 (R^2^ of 0.99; Fig. 1d).


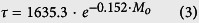


The parameter *k* represents the water absorption rate of the process. The higher the initial moisture content of the grain was, the higher the value of this parameter was; however, in a more complex pattern. It has a constant value at lower initial moisture content of the grain since the main entrance of water is the hilum, limiting the hydration rate. However, when the beans reach ~23% d.b, the *k* value sharply increases. From this moisture content, the seed coat is very permeable to water, allowing the water enters not only by the hilum, but also by the seed coat, which involves the increment of the value of this parameter. Further, when the initial moisture content of the beans is close to the equilibrium moisture content (very high), the value of the parameter *k* is reduced until constant rate. This happens probably because of the mass transfer driving force (water activity difference) is reduced, reducing the hydration rate. In this case, a sigmoidal model was used to explain the behavior of this parameter (Equation 4; R^2^ of 0.97; Fig. 1e).


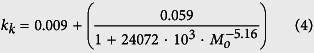


**Figure 3:**
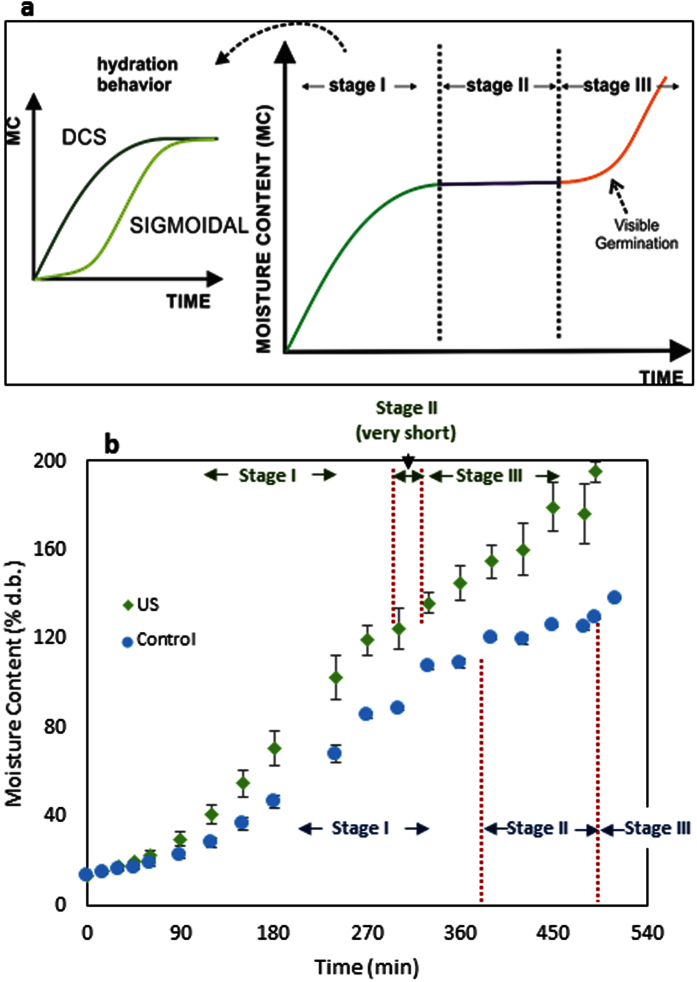
(**a**) Stages of the seeds germination as function of the moisture content. Adapted from Bewley and Black^30^ and ref. 18. (**b**) Ultrasound assisted hydration process increases the hydration and germination velocity of mung bean. The dots are the experimental values and the vertical bars are the standard deviation.

Finally, the equilibrium moisture content parameter (*M*_∞_) value decreased as the initial moisture content increased. However, this result was not presented for Adzuki beans^21^ nor lentils^26^. This can be explained according the following hypothesis. Mung bean is characterized by a fast germination^35^, and due to the germination enzymes are more active at relative high initial moisture content (up to 20% d.b. of moisture content the enzyme are activated^36^), the radicle growth could start earlier. Therefore, if the radicle starts to grow faster, additional water will be absorbed, and the stage I will finish earlier reducing the equilibrium moisture content of this stage (see the section 2.4; Fig. 3a). Beans with high initial moisture content have the moisture homogeneously distributed in the bean. Thus, enzymes are more active in whole bean, triggering the germination process (radicle growth). In contrast, beans, which reach high moisture contents by the hydration process of dry beans (12.25% d.b. of moisture content), have a heterogeneous distribution of the moisture, having a higher moisture content in the external parts and a lower moisture content in the internal parts of the bean. Consequently, the enzymes of the internal layer of the embryo are not activated, delaying the germination process until the complete hydration of the bean. Due to the observed pattern, the equilibrium moisture content was fitted to a composed exponential equation (Equation 5; R^2^ of 0.99; Fig. 1f).


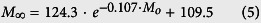


### Ultrasound assisted hydration of mung beans and impact on its germination

Mung bean can be used as a grain or as a seed, depending of its finality. When the germination process is involved, mung bean can be considered as a seed; when germination is not involved, it is a regular pulse and grain^37^.

During the germination process, the seed hydration follows a tree-stage water uptake pattern (Fig. 3a). The first stage consists of the hydration process itself (as described in sections 2.1), when the seed absorbs the necessary water to activate its metabolism. In this stage, the seed arises the first signs of metabolism reactivation^37^. The second stage consists of reserves digestion and new molecules synthesis. In this stage, the hydration of the seed is negligible (it can be considered as the equilibrium moisture content of the hydration process of grains, i.e., the *M*_∞_ of stage I – as described above). Stage III takes place when the radicle starts to grow and many structural components are synthetized; thus, water is required in many metabolic processes, resulting in more water absorption^37^. Therefore, the hydration process in the third stage is mainly due to biological phenomena.

In the case of grains, used as food, only stage I is important for their processing. Thus, during the hydration study of grains, only the stage I is evaluated (which can be widely observed in the literature). However, in the present work, although the hydration modeling (section 2.5) was conducted only in the stage I, the process was evaluated until stage III, when a small, but visible radicle proves the start of germination. Further, as previously described, the food hydration does not have only a DCS behavior, but in some cases, it also shows a sigmoidal behavior. Therefore, Fig. 3a was complemented, highlighting the two possible hydration behavior at stage I. As mung bean has a short phase II, of approximately 2 h^35^, the germination process is very fast. This may explain the reduction of the equilibrium moisture content when a bean with high initial moisture contents were hydrated. At high initial moisture contents, the beans are metabolically more active, reducing the minimum moisture content to germinate and the length of stage I and stage II.

Figure 3b shows the effect of the ultrasound technology (41 W/L, 25 kHz of frequency) on the hydration process of mung beans. It can be clearly seen that the ultrasound enhanced the hydration process, reducing approximately 25% of the time to reach the equilibrium moisture (i.e., the stage I duration, reaching the stage II). Besides this successful result, ultrasound also accelerated the germination process of this bean by reducing the stage I length and almost disappearing the stage II.

In fact, the ultrasound has improved the hydration process of other grains such as chickpeas^14^,^38^, navy beans^13^, sorghum grains^12^, common beans^15^, and corn kernels^16^. Most of these works attributed the improvement to the direct and indirect effects of ultrasound on mass transfer processes^9^ – strictly physical mechanisms of mass transfer improvement.

However, it was demonstrated that the ultrasound technology enhances the seeds vigor, probably by enhancing its metabolism^39^. Consequently, the hydration process may also be enhanced not only by physical phenomena, but also due to metabolic/biological phenomena (also accelerating the germination). In fact, although it was still not described, it is a possibility that can explain the observed behavior. In fact, this possibility must be further evaluated; unfortunately, it cannot be proved in the present work.

At stage I, the moisture content of the beans is low and, consequently, the water activity too. Therefore, the enzyme activity in the grain is also low, being increased when the moisture content increases. In this part of the process, the main improvement by the ultrasound technology may be physical, due to its direct and indirect effects. The direct effects are the inertial flow and the sponge effect, which by taking advantage of the porosity of the bean, increases the water intake by pumping the water into the tissues and by unblocking the pores^9^,^16^. In addition, the traveling of the ultrasonic waves probably caused the change of the beans pores size or shape. As the beans moisture content is increased, probably the indirect effects gain strength, since the water vapor is increased, facilitating the acoustic cavitation and the formation of micro cavities and micro-channels^9^. Consequently, both the ultrasonic direct (inertial flow and sponge effect) and indirect effects (micro-channels formation) could take place at the final part of the stage I, improving the hydration process.

**Figure 4:**
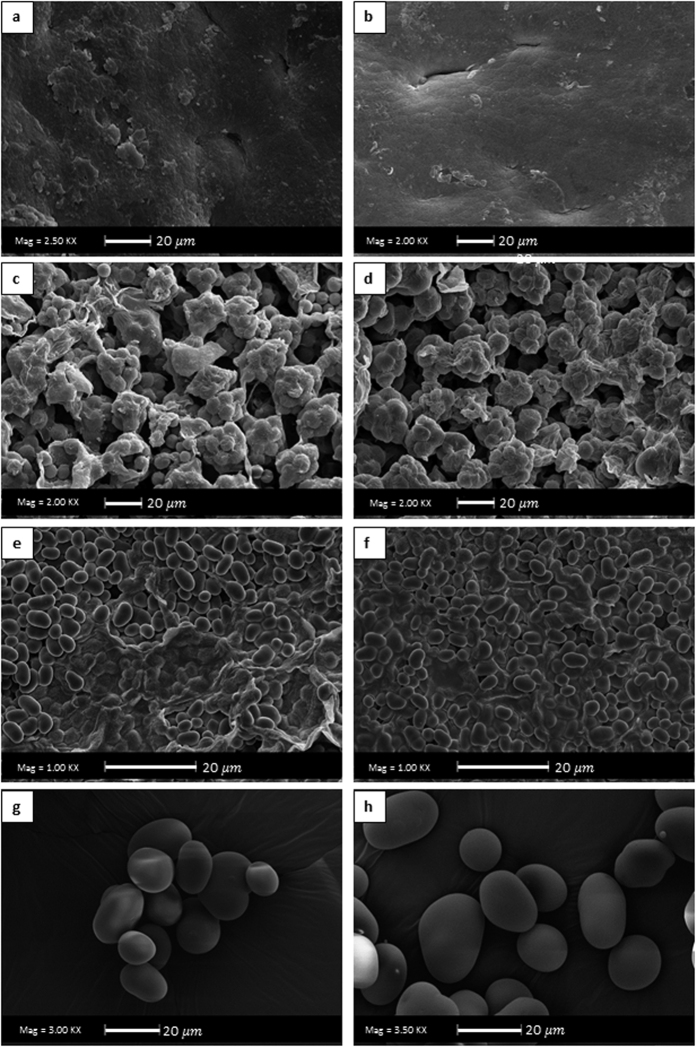
Microphotography SEM (20 kV; the magnifications are shown in the figures) of the different structures and starch of mung bean. (**a**,**c**,**e** and **g**) are the seed coat surface, hilum, cotyledon and starch, respectively, from conventionally hydrated beans. (**b**,**d**,**f** and **h**) are the seed coat surface, hilum, cotyledon and starch, respectively, from from ultrasound assisted hydrated beans.

Figure 4 shows the microstructure of mung bean hydrated with and without ultrasound. There was not any significant visible difference among the structures of the bean (seed coat, hilum and cotyledon) hydrated with and without ultrasound. In addition, Fig. 4g and h demonstrated that the structure of the starch was not modified (the effect of ultrasound on the mung bean starch is discussed in the following section). In conclusion, as other previous works^9^,^16^, it is demonstrated that the ultrasound technology (at the used conditions of power and frequency) did not cause significant changes on the grains structure. Although the micro-channels formation was demonstrated for sorghum grains^9^, the Scanning Electronic Microscopy probably is not a suitable analysis for detecting the formed micro-channels. Probably, the micro-channels are too small or difficult to identify. In addition, the sample preparation of this technique (as the grain must be dried, which definitely affects its structure) could have more effect on the microstructure than the process, which hinders the possible changes that ultrasound could have caused. Therefore, other techniques could be studied in future researches. However, SEM analysis gave us an idea that ultrasound did not change the overall structure, and that the modifications are slight.

Furthermore, the ultrasound reduced the stage II (Fig. 3a and b), causing the bean germination, leading to the stage III. In fact, the ultrasound technology has improved the germination process of other seeds, such as barley^39^,^40^, switchgrass^41^, pea^42^ and grass seeds^43^. Most of those works gave as the possible effect of ultrasound on the germination process, the increasing in the nutrient mobility, the respiration rate and/or the water availability for metabolic reactions. During stage II the reserve components digestion takes place, as well as the nutrient transport and the synthesis of some components^37^. Therefore, ultrasound could have improved those processes for mung bean, helping the reserve molecules catabolism and the transport of molecules to the radicle (mass transfer improvement), reducing the stage II duration. In fact, Liu *et al*.^43^, demonstrated that the ultrasound technology increased the metabolic activity of aged grass seed, enhancing the germination percentage, attributing this improvement to the cited reasons and the increment of the porosity of the seed by the acoustic cavitation. In addition, the vibration caused by ultrasound could have caused the increment of the metabolism activity, accelerating the germination process as it was demonstrated that a sinusoidal vibration enhances the germination process^44^.

It is interesting to highlight that the acceleration of the mung bean germination is a desirable result, as this grain is widely consumed as a sprout. Consequently, the ultrasound technology can be useful for the mung bean sprout (also called as Moyashi) production, by accelerating both the hydration and germination.

### Ultrasound assisted hydration of mung beans: impact on mass transfer and modeling

**Figure 5:**
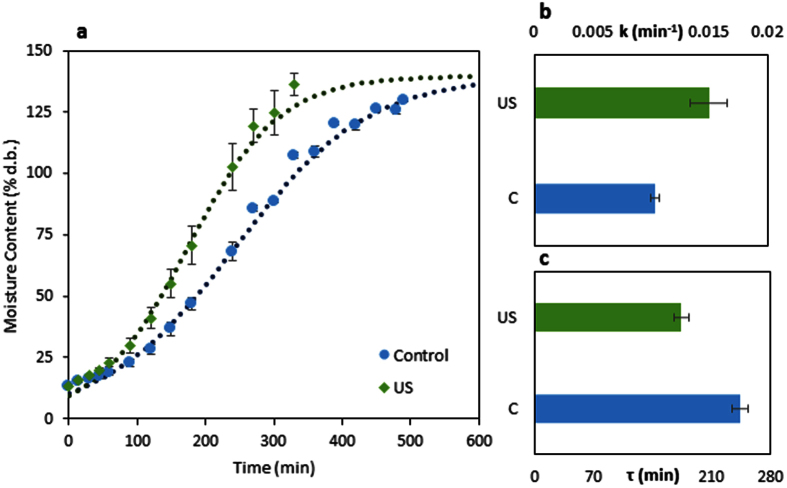
(**a**) Mathematical modeling of the hydration process with and without the ultrasound technology. The dots are the experimental values; the bars are the standard deviation and the curves are the model values. (**b**) Effect of the ultrasound technology on *k* parameter of Kaptso *et al*. model (Equation 2). (**c**) Effect of the ultrasound technology on *τ* parameter of Kaptso *et al*. model (Equation 2).

Finally, the hydration process with and without ultrasound was modeled using the Kaptso *et al*. model^8^ and its parameters were evaluated (Fig. 5). It should be mentioned that for the ultrasound assisted hydration, the data of the phase III were not considered, using the data until the beginning of stage II since this model only describes the hydration process (stage I). Therefore, the value of the equilibrium moisture content (*M*_∞_) was fixed and considered the same to the control treatment. Despite this consideration, the Kaptso *et al*. model successfully fitted the experimental data (R^2^ of 0.99 for both treatments). The parameters *k* and *τ* had significant difference (p < 0.05) when ultrasound was applied for the hydration process.

The parameter *k*, which is related to the hydration rate, increased when the beans were hydrated with ultrasound, from 0.0104 ± 0.0004 min^−1^ to 0.0150 ± 0.0016 min^−1^ (an increase of ~44%). It means that the ultrasound decreases the internal resistance for the water flow through the bean. As described, this enhancement could be probably caused by the direct effects at the first part of the process (due to the low moisture content of the beans), and probably by both direct and indirect effects at the final part of the hydration process (due to the high moisture content of the beans), increasing the total hydration rate.

The parameter *τ*, which is related to the lag phase of the hydration process, decreased almost 28% when the ultrasound technology was applied, from 243.7 ± 9.6 min to 174.5 ± 14.2 min. The lag phase of the hydration process ends when the seed coat is enough hydrated to increase its permeability^21^,^26^. Therefore, the ultrasound technology caused the rapid entry of water in the first part of the process, hydrating faster the seed coat, increasing its permeability and accelerating the hydration process. It is very likely that this improvement has been due to the direct effects, helping the lateral hydration of the bean through the space between the seed coat and the cotyledon and through the osteosclereids cells of the seed coat.

There is not any work in the literature relating the ultrasound-assisted hydration of sigmoidal behavior hydration beans. Thus, it is the first work that demonstrated that ultrasound reduces the lag phase of the hydration process. However, further studies should be performed to determine whether higher power ultrasound could further reduce the lag phase.

All these results demonstrate that the ultrasound is a promising technology, which can be implemented in the industries since it reduces the hydration process time and, in some cases, could accelerate the germination process of seeds, which is very desirable for the sprouting and malting process.

### Effect of the ultrasound assisted hydration on the properties of mung bean flour and starch

**Figure 6:**
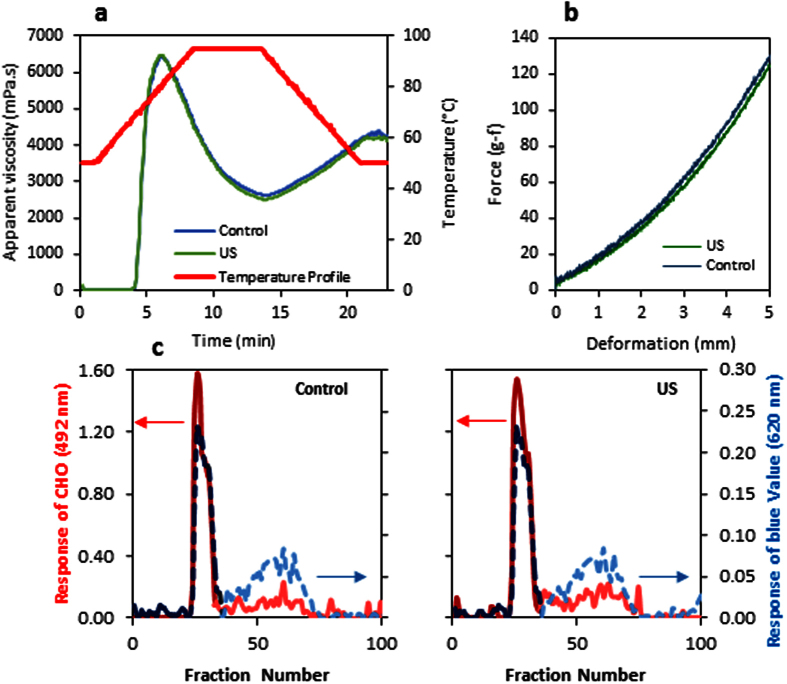
Evaluation of the starch properties extracted from mung beans hydrated without (Control) and with ultrasound (US). (**a**) RVA profile. (**b**) Texture of the starch gel. (**c**) Sepharose CL 2B gel permeation chromatograms: Continuous red curves represent the response of CHO and the dot curves represent the response of blue value. In addition, darker colors represent the higher molecular weight region (amylopectin) and lighter colors represent the lower molecular region (amylose).

Figure 4g and h shows the SEM microphotographs of the isolated starches from the beans hydrated without and with ultrasound, respectively. Both shows an oval to spherical shaped with smooth surface without fissures as verified by Rupollo *et al*.^45^. Therefore, ultrasound did not change the structure of the starch grain. In some works were shown that ultrasound changed the starch microstructure modifying its technological properties^46^,^47^. In addition, the Rapid Viscosity Analysis (RVA) profile of the starch suspensions (Fig. 6) also demonstrated that the ultrasound technology did not alter the pasting properties of starch isolated from the hydrated beans (for all the evaluated parameters: peak, trough, breakdown, setback and final apparent viscosity), reinforcing the results of SEM. This result was different in comparison with those carried out using isolated starch granules in suspension, such as the work of Zuo *et al*.^48^. They demonstrated that the ultrasound technology reduces the apparent viscosity of the starch suspensions. However, it is necessary to clarify that in the mentioned works, the results obtained in the SEM and RVA analysis were acquired with isolated starches treated with ultrasound, in contrast to the present work, where the starch was still inside the grains (cotyledon), fiscally protected, when the ultrasound technology was applied. This result is also in accordance with the work of Miano^16^.

Figure 6 also shows the force-displacement graphic obtained from the gel texture evaluation. The gel strength is associated with starch constituents (amylose and amylopectin) and the interaction between them^49^. Therefore, any changes in the starch gel texture (keeping constant all the other parameters, such as temperature, concentration, etc.) would be due to the molecular depolymerization and amylose molecular size reduction, which are directly associated with starch retrogradation and its ability to form gels^50^,^51^. There was not significant change (p < 0.05) between the gel strength of the starch isolated from the mung bean hydrated without and with ultrasound. This means, therefore, that there was not significant change in the starch molecular structure when the beans were hydrated using the ultrasound technology.

Finally, Sepharose CL 2B gel permeation chromatograms of bean starches are shown in Fig. 6. The first peak corresponds to amylopectin and the second one (determined by the blue value of the iodine) corresponds to amylose^52^,^53^. These results suggested any important change in the molecular weight and structure of the starches isolated from mung beans hydrated with and without ultrasound – reinforcing the previous results.

The obtained results demonstrate that the ultrasound technology did not affect both the starch structure and technological properties during mung bean hydration process, which is highly relevant for the starch industry.

**Figure 7:**
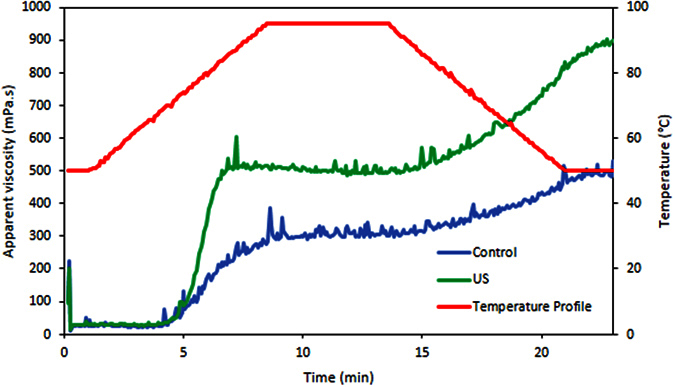
Rapid viscosity analysis profile (RVA) of the flour of mung beans hydrated with (US) and without (Control) ultrasound.

On the other hand, the RVA profile of the beans flour (Fig. 7) demonstrated that the ultrasound caused an increment of the apparent viscosity. Higher apparent viscosity could be beneficial for some food industries, considering bean based products that need higher consistency. Similar results were obtained by Ghafoor *et al*.^13^ who demonstrated that the apparent viscosity of flour from navy beans hydrated with ultrasound was higher than the hydrated without ultrasound. However, they attributed this change to the starch modification by ultrasound, even though, that work has only evaluated the flour. Nevertheless, as the present work performed the RVA profiles of both starch and flour, and as there was not any difference in the starch structure and properties (Fig. 6), it can be demonstrated that the ultrasound changed the protein properties, instead of the starch properties (Fig. 7). Ultrasound technology might have altered the beans protein structure, modifying the accessibility of water molecules to the binding sites of the protein chains. O’Sullivan *et al*.^54^ observed that the ultrasound reduces the aggregates size of legume protein increasing the solubility of them^55^,^56^. Therefore, the proteins solubility increasing can explain the increment of the beans flour apparent viscosity.

## Conclusions

Mung bean hydration process has a sigmoidal behavior and, similarly to other pulses, this behavior changed to the Downward Concave Shape behavior when the initial moisture of the grain is approximately 23% d.b. The route of water entrance of this bean was established according to the seed coat permeability and the water absorption participation of the hilum and the seed coat. Furthermore, it was demonstrated that the ultrasound technology improved the hydration process of mung bean, reducing the total process time almost 25% (reducing the lag phase time ~28% and increasing the water absorption rate ~44%). In addition, it was demonstrated that this technology accelerated the germination process of this bean, which is a very desirable result for sprouting or malting. Finally, it was concluded that the ultrasound technology did not alter the starch properties (structural and rheological). However, this technology increased the apparent viscosity of the whole bean flour, which was attributed to the proteins changes. Considering everything, the ultrasound technology can be used to accelerate the hydration process of this bean without altering its starch. In addition, depending on the purpose, the ultrasound can be used to accelerate the germination process, being these results very desirable for pulses industry.

## Materials and Methods

### Raw Material

Mung bean (*Vigna radiata;* 12.25 ± 0.53% d.b (g water/100 g of dry matter) of moisture content; 5.12 ± 0.24 mm length, 3.81 ± 0.22 mm width and 3.62 ± 0.16 mm thick) obtained at a local market of Campinas - Brazil was used.

### Conventional hydration process description

For the hydration process, 10 g of pre-selected grains (without any damage) were placed into net bags and soaked in 4 L of distilled water (to avoid water be a limiting in the process) at 25 ± 1 °C during all kind of treatments. During the hydration process, the grains were periodically drained, superficially dried and their moisture content were obtained by mass balance using the initial moisture content (determined using a Moisture Analyzer MX-50 AND, Japan) (after verifying the possibility to neglect the solid loss to the water). Then, the grains were soaked again to continue the process. The grains were weighted every 15 min for the first hour, every 30 min for the latter two hours and every hour from then on. The hydration process was performed at constant temperature using a water bath (Dubnoff MA 095 MARCONI, Brazil) and in triplicate.

### Effect of the initial moisture content on the hydration behavior

To study the effect of the initial moisture content on the hydration behavior, generating subsidies for better understand the hydration mechanisms, samples with different initial moisture content were prepared. To obtain samples with a higher initial moisture content (15.83, 18.95, 23.63, 35.29 and 41.95% d.b.) the grains were hydrated for a specific time at 25 ± 1 °C. Then, these grains were put into sealed containers for a week at 5 ± 1 °C to homogenize the moisture into the grains. The lower initial moisture content sample (3.88% d.b.) was prepared by placing the grains in a desiccator with silica gel for 2 weeks until obtaining the required moisture content. The initial moisture content of the samples was then obtained by a mass balance using the moisture content of the original sample^21^.

Further, the sorption isotherm was also elaborated. Beans with different moisture contents (prepared using the procedure described above) were ground using a cutter mill prior to determining their water activity at 25 °C using a water activity meter (AquaLab 4TE, Decagon Devices, Inc USA). The sample moisture content (% d.b.) was then plotted as function of the water activity. The obtained curve was modeled using the Oswin Equation (Equation 6) since it is recommended for starchy food^29^. In this equation, M is the moisture content of the product (% d.b.), *a*_*w*_ is the water activity of the product and A and B are model parameters related to the curve shape.


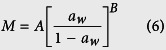


### Study of the water entrance route

In this case, the hydration process was performed with some covered structures. In addition, the microstructure of the grains was studied to observe the different structures of the grain (according to Fig. 2).

To verify the water entrance, the seed coat or the hilum were covered using a varnish (nail polish; Risqué – Cosmed Industry Brazil) as a sealant, similar to Ramos *et al*.^57^. This treatment allowed determining the contribution of these structures to the hydration process.

For the microstructural analysis, the samples were cut with a scalpel blade to see the different tissues (seed coat, cotyledon, and external surface) and dehydrated in a sealed container using silica gel for 3 days. Then, they were sputtered with a 30 nm gold layer. Finally, the samples were observed in a scanning electronic microscope operated at an acceleration voltage of 20 kV (LEO 435 VP, Leo Electron Microscopy Ltd., Cambridge, England).

### Ultrasound assisted hydration

During the experiments, an ultrasonic bath with a frequency of 25 kHz and a volumetric power of 41 W/L (Q13/25, Ultronique Brazil; determined following the method described by Tiwari *et al*.^58^) was used. This bath has its piezoelectric elements arranged below the tub. It generates the mechanical waves that are transmitted through the water to the product. The ultrasonic waves distribution in the water bath was determined by the method of the aluminum foil^59^,^60^. Further, the other good practices described by^59^,^60^ were also verified. Thus, the samples were placed in the parts where the waves had the highest and most homogeneous intensity.

The ultrasound-assisted hydration was performed in the ultrasonic water bath with 4 L of water at 25 ± 1 °C. The grains were placed into net bags and placed on the bottom of the ultrasonic water bath. The data were collected as the control hydration process explained above.

### Modeling of the hydration process

The Mung bean hydration kinetics was modeled using the sigmoidal equation of Kaptso *et al*. (Equation 2; ref. 8). For that purpose, the dry basis moisture content of the grains (M% d.b.) versus the hydration time (min) was tabulated for each initial moisture. The data were fitted to the mathematical model with a confidence level of 95% using the Levenberg-Marquardt algorithm in Statistica 12.0 (StatSoft, USA) software.

Finally, the goodness of fit of the models was evaluated by the R^2^ regression value, the root-mean-square deviation values (RMSD, Equation 7), the normalized RMSD (NRMSD, Equation 8) and by plotting the moisture content values obtained by the model (M_model_) as a function of the experimental values (M_experimental_). The regression of those data to a linear function (Equation 9) results in three parameters that can be used to evaluate the description of the experimental values by the model, i.e. the linear slope (a, which must be as close as possible to one), the intercept (b, which must be as close as possible to zero) and the coefficient of determination (R^2^; that must be as close as possible to one).


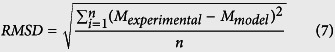



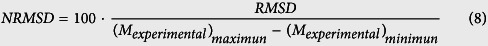



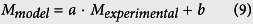


### Starch and flour evaluation

The starch of the mung beans (hydrated with and without ultrasound) was extracted as follow: The hydrated beans (with and without ultrasound) were milled (with distilled water) using a blender and sieved (60 and 325 mesh). The supernatant was washed two times with distilled water. The filtrate was centrifuged at 3200 g for 5 min, for them separating the starch from the rest of the components (water, proteins and lipids). Finally, the starch was dried at 35 °C for 12 h in a flat tray and was softly milled using a mortar and pestle.

The flour of the mung beans (hydrated with and without ultrasound) was obtained by grinding them after the hydration process using a cutter mill.

To evaluate if the obtained starch or flour was affected by the ultrasound, the following evaluation was performed.

The mung bean flour (i.e., the whole grain milled) and starch pasting properties were evaluated in a Rapid Visco Analyzer (RVA-S4A; Newport Scientific, Warriewood, NSW, Australia) using 3 g of sample (corrected for 14% of moisture) in 25 g of water. The suspension was first held at 50 °C for 1 min and then heated to 95 °C at a rate of 6 °C · min^−1^. The sample was then held at 95 °C for 5 min, followed by cooling to 50 °C at a rate of 6 °C · min^−1^, and finally holding it at 50 °C for 2 min. As the starch was evaluated separately from the flour, the differences between their rheological profiles could be related with the changes on the product proteins (the two main components of the grain: 31.1% of starch^61^ and 23.8% of protein^20^ as average).

Microstructure of the starch was evaluated using scanning electronic microscopy in similar way as the beans analysis. The starch was placed on the stubs using a dry brush and passing directly to the sputtering process.

The mechanical properties of the starch gel were also analyzed by instrumental texture. The gel strength was determined using a Texture Analyzer (TA.XT Plus, Stable Micro Systems Ltd., Surrey, UK) with a load cell of 5 kg-f (49,03 N). The gel obtained after the RVA determination was stored in a 40 × 20 mm (diameter × height) plastic cup for 24 h at room temperature to stay solid before evaluation. To ensure uniform moisture of the samples, they were held in a desiccator with water at the bottom. A 0.5 cm cylindrical probe (P/0.5 R) was used to compress the samples until the distance of 5 mm at 1 mm s^−1^. The force measured by the equipment as a function of the penetration depth was then used to evaluate the gel strength.

The molecular mass distribution profiles of the starch samples were determined by gel permeation chromatography, using a GE XK 26/70 column (2.6 cm diameter and 70 cm high), packed with Sepharose CL-2B gel (Sigma, Sweden). 10 mL of dimethylsulfoxide (DMSO; 90%, Labsynth, Brazil) was added to 0.1 g of starch and heated in boiling water bath for 1 h, then remaining for 24 h at 25 °C under constant stirring. An aliquot of 3 mL (30 mg of starch) was then mixed with 10 mL of absolute ethanol to precipitate the starch, being the suspension centrifuged for 30 min at 3000 g. The precipitated starch was dissolved in 9 mL of boiling distilled water and put in boiling water bath for 30 min^52^. An aliquot of 4 mL was then eluted in the chromatographic column upwardly. A solution containing 25 mmol · L^−1^ of NaCl and NaOH 1 mmol · L^−1^ was used as eluent at a rate of 60 mL · h^−1^. Fractions of 4 mL were collected (Gilson model FC203B, Middleton, England) and analyzed for total carbohydrate content at 490 nm by the phenol sulfuric method (Dubois *et al*., 1956) and blue value at 620 nm (Juliano, 1971), using a microplate reader (Asys Expert plus, Biochron, England).

### Statistical evaluation

When relevant, statistical analysis was performed to the treatments through analysis of variance (ANOVA) and Tukey’s test (P ≤ 0.05), using the software Statistica 12.0 (StatSoft, USA).

## Additional Information

**How to cite this article**: Miano, A. C. *et al*. Enhancing mung bean hydration using the ultrasound technology: description of mechanisms and impact on its germination and main components. *Sci. Rep.* **6**, 38996; doi: 10.1038/srep38996 (2016).

**Publisher's note:** Springer Nature remains neutral with regard to jurisdictional claims in published maps and institutional affiliations.

## Supplementary Material

Supplementary Information

## Figures and Tables

**Table 1:** Parameter values from the mathematical model that evaluate the effect on the initial moisture content on the hydration kinetic of Mung bean (mean ± standard deviation).

Initial Moisture Content (% d.b.)	τ (min)	k (min^−1^)	M_∞_ (% d.b.)	R^2^	RMSD (% d.b.)	NRSMD (%)
12.25	252.2 ± 4.7	0.0099 ± 0.0003	143.5 ± 1.7	0.99	1.0	0.8
15.83	148.0 ± 2.0	0.0128 ± 0.0003	131.1 ± 0.5	0.99	3.0	2.7
18.95	95.5 ± 3.3	0.0165 ± 0.0006	126.2 ± 0.9	0.99	3.4	3.2
23.63	38.1 ± 1.9	0.0270 ± 0.0010	120.3 ± 2.5	0.98	7.6	8.6
35.29	7.6 ± 0.1	0.0724 ± 0.0017	111.8 ± 0.8	0.96	13.3	18.8
41.95	5.0 ± 0.2	0.0681 ± 0.0028	111.1 ± 0.4	0.96	12.2	18.8
