# Supplementary material for: Enhancing mung bean hydration using the ultrasound technology: description of mechanisms and impact on its germination and main components
Source: Sci Rep. 2016 Dec 19;6:38996. doi: 10.1038/srep38996 (PMC5171542; doi:10.1038/srep38996)
Supplement: Supplementary Information [file srep38996-s1.pdf]

SUPLEMENTARY INFORMATION

Enhancing mung bean hydration using the ultrasound technology: description of mechanisms and impact on its germination and main components

*Alberto Claudio Miano, Jessica da Costa Pereira, Nanci Castanha, Manoel Divino da Matta Júnior and Pedro Esteves Duarte Augusto**

Table. Analysis of the starch extracted from mung beans hydrated conventionally and with ultrasound (mean values ± standard deviation). The letters next to the values represent the mean comparison test ($p<0.05$)

RVA Analysis	Conventional hydration	Ultrasound
Peak 1 (mPa.s)	6520 ± 107[a]	6321 ± 84[a]
Trough 1 (mPa.s)	2634 ± 16[a]	2436 ± 26[a]
Breakdown (mPa.s)	3886 ± 123[a]	3885 ± 58[a]
Final apparent viscosity (mPa.s)	4187 ± 7[a]	4115 ± 182[a]
Setback (mPa.s)	1553 ± 9[a]	1680 ± 209[a]
Peak Time (min)	6.1 ± 0.1[a]	6.1 ± 0.1[a]
Pasting Temperature (°C)	67.6 ± 0.2[a]	67.4 ± 0.4[a]
